# Progress in the Clinical Application of Artificial Intelligence for Left Ventricle Analysis in Cardiac Magnetic Resonance

**DOI:** 10.31083/j.rcm2512447

**Published:** 2024-12-19

**Authors:** Yinghui Le, Chongshang Zhao, Jing An, Jiali Zhou, Dongdong Deng, Yi He

**Affiliations:** ^1^Department of Radiology, Beijing Friendship Hospital, Capital Medical University, 100050 Beijing, China; ^2^Key Laboratory for Biomedical Engineering of Ministry of Education, Institute of Biomedical Engineering, Zhejiang University, 310058 Hangzhou, Zhejiang, China; ^3^Siemens Shenzhen Magnetic Resonance, MR Collaboration NE Asia, 518000 Shenzhen, Guangdong, China; ^4^School of Biomedical Engineering, Dalian University of Technology, 116024 Dalian, Liaoning, China

**Keywords:** cardiovascular magnetic resonance, artificial intelligence, left ventricle

## Abstract

Cardiac magnetic resonance (CMR) imaging enables a one-stop assessment of heart structure and function. Artificial intelligence (AI) can simplify and automate work flows and improve image post-processing speed and diagnostic accuracy; thus, it greatly affects many aspects of CMR. This review highlights the application of AI for left heart analysis in CMR, including quality control, image segmentation, and global and regional functional assessment. Most recent research has focused on segmentation of the left ventricular myocardium and blood pool. Although many algorithms have shown a level comparable to that of human experts, some problems, such as poor performance of basal and apical segmentation and false identification of myocardial structure, remain. Segmentation of myocardial fibrosis is another research hotspot, and most patient cohorts of such studies have hypertrophic cardiomyopathy. Whether the above methods are applicable to other patient groups requires further study. The use of automated CMR interpretation for the diagnosis and prognosis assessment of cardiovascular diseases demonstrates great clinical potential. However, prospective large-scale clinical trials are needed to investigate the real-word application of AI technology in clinical practice.

## 1. Introduction

Cardiovascular disease remains the leading cause of mortality and morbidity worldwide [1]. Cardiovascular magnetic resonance (CMR) is a non-invasive method of evaluating cardiac morphological and histological changes and plays an increasingly important role in diagnosing cardiovascular diseases [2]. Guidelines indicate that a complete CMR imaging diagnostic report should include left ventricular end-diastolic volume (EDV), end-systolic volume (ESV), stroke volume (SV), ejection fraction (EF), cardiac output, left ventricular (LV) mass, LV myocardial thickness, LV wall motion, and late gadolinium enhancement (LGE) [3]. However, calculating and analyzing these indicators is time-consuming and requires great expertise by radiologists. Additionally, differences exist between observers, which may affect accurate patient assessments.

**Fig. 1.**
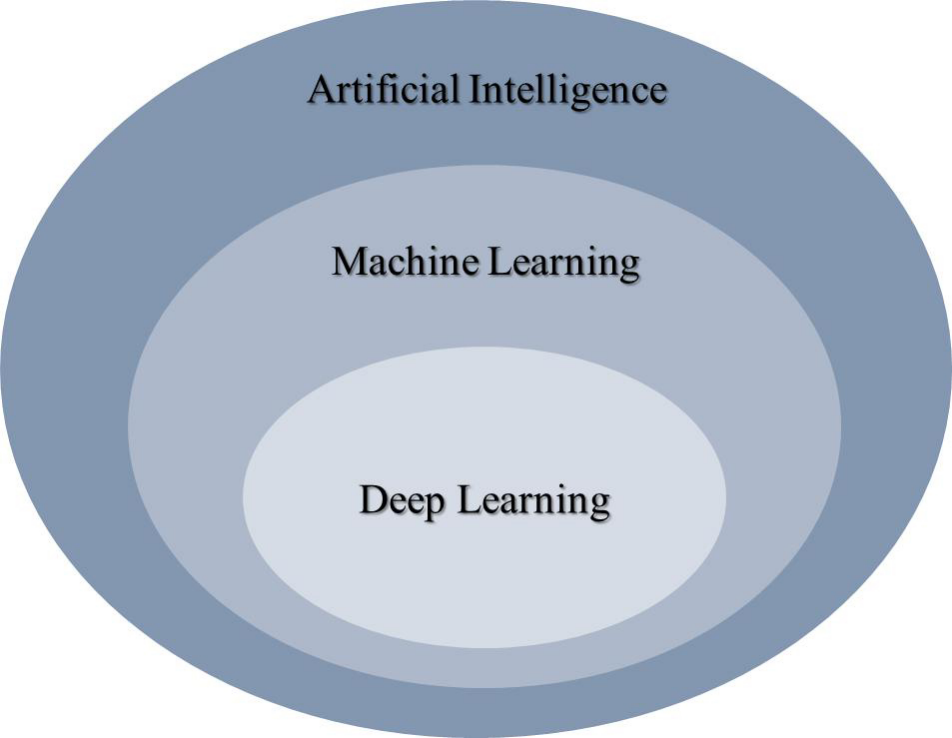
**Diagram of the relationship between artificial intelligence (AI), machine learning (ML) and deep learning (DL)**.

In recent years, the medical industry has entered an era of digital intelligence, and the deep integration of artificial intelligence (AI) and medical imaging may soon be possible. An advantage of AI is that it can complete simple manual repetitive work through training models and improve efficiency and observer consistency [4, 5]. AI refers to the broad field of computer science focused on creating machines that can perform tasks requiring human-like intelligence. These tasks may include understanding natural language, recognizing patterns, making decisions, and solving problems. Fig. 1 shows the relationship between AI, machine learning (ML) and deep learning (DL). ML is a subset of AI that involves developing algorithms and statistical models that enable computers to learn from and make predictions or decisions based on data. ML algorithms can be subdivided into supervised, unsupervised, and reinforced learning. In supervised learning, the model is trained on a dataset that includes both input data and corresponding correct output labels. In unsupervised learning, the model is trained on input data without labeled data. In reinforcement learning, the objective is to maximize cumulative reward over time by learning optimal strategies or policies [6]. Deep learning is a branch of ML that draws inspiration from the structure and function of the human brain, specifically through artificial neural networks. DL aims to interpret data by simulating algorithms that mimic human brain analysis and learning mechanisms [7]. Although ML and DL have been used in image acquisition and reconstruction, image segmentation, cardiovascular disease diagnosis and risk assessment, there are still great challenges [8]. As shown in Table 1 (Ref. [5, 9, 10, 11, 12, 13, 14, 15, 16, 17, 18, 19, 20, 21, 22, 23, 24, 25, 26, 27, 28, 29, 30, 31, 32, 33, 34, 35, 36, 37]). This review summarizes and analyzes the current application progress and limitations of AI in analyzing LV structure and function. We searched and reviewed studies on AI related to the left ventricle to provide guidance and suggestions for future research.

**Table 1.** **Summary of AI techniques, applications, and challenges used in the analyzed studies**.

Study	Method	Image substrate	Application	Challenge
[5, 9]	FCN	Cine	Biventricle segmentation	◆ The boundary between trabeculation and myocardium was blurred.
[10]	NCDN	Cine	Biventricle segmentation	◆ Inclusion of the atrium in slices close to the base, with intensities similar the internal region of the LV.
[11]	DRN	Cine	Biventricle segmentation	◆ Similar intensity between the myocardium and nearby regions, which can hinder epicardial extraction.
[12]	CNN	Cine	Biventricle segmentation	◆ Different vendors have differences in image quality.
[13, 14]	CNN	Cine	LV segmentation	◆ The lack of adequate high-quality of annotation datasets.
[15]	nnU-Net	Cine	Biventricle segmentation and quality control	
[16]	FCDA-Net	Cine	LV segmentation	
[17]	MMNet	Cine	LV segmentation	
[18]	CNN, U-Net, RNN	Cine	Landmark detection	
[19]	CNN	Cine	Landmark detection	
[20]	CNN	Cine	Myocardial thickness	
[21]	RNN, FCN	Cine	Biventricle segmentation and myocardial motion	
[22, 23]	CNN	Cine	Myocardial motion	
[24]	FCN	Cine	Myocardial motion	
[25, 26]	RNN	Cine	Myocardial motion	
[27, 28]	CNN	LGE	Myocardial fibrosis	◆ Images with complex contrast and noise.
[29]	FCN	LGE	Myocardial fibrosis	◆ The lack of adequate high-quality of annotation datasets.
[30]	ACSNet	LGE	LV segmentation and myocardial fibrosis	◆ The fusion of cine with LGE is difficult.
[31]	CNN	Cine, LGE	Myocardial fibrosis	
[32]	CNN	Cine, T1 mapping	Myocardial fibrosis	
[33]	DL	Cine	Myocardial fibrosis	
[34]	ML	Cine	Myocardial fibrosis	
[35]	DL	Cine	Quality control	◆ Image quality is influenced by many factors.
[36]	CNN	T1 mapping	Quality control	◆ The image features of different sequences and vendors are different.
[37]	CNN	Cine	Biventricle segmentation and quality control	

FCN, fully convolutional network; NCDN, nested capsule dense network; DRN, dilated residual network; CNN, convolutional neural network; FCDA-Net, fine, grained calibrated double, attention convolutional network; MMNet, multi, scale multi, skip connection network; RNN, recurrent neural network; ACSNet, anatomical convolutional segmentation network; DL, deep learning; ML, machine learning; LV, left ventricular; LGE, late gadolinium enhancement; AI, artificial intelligence; nnU-Net, no-new-Net.

## 2. LV Segmentation

Accurately identifying the cardiac structure is a prerequisite for obtaining accurate EDV, ESV, EF and other cardiac function parameters. In recent decades, automatic or semiautomatic computer methods for analyzing CMR have been developed to achieve automatic and accurate segmentation of the LV myocardium (LVM) and LV blood pool (LVBP).

### 2.1 Methods and Robustness

Existing LV segmentation algorithms primarily include five categories: atlases, graphs, deformable models, image-based, and AI. Approximately half of these studies (44.6%) employed AI to identify the LV [4]. Of these, 75.7% used DL [4]. Among all DL architecture, the most mature and widely used is the convolutional neural network (CNN), represented by the fully convolutional network (FCN). The U-Net is the most used and explored FCN in the literature. Several modifications for the U-Net including structural changes are produced [7]. Studies have shown that the combination of U-Net and transformer could greatly reduce the difficulty and improve the accuracy of segmentation. The various networks have differing performance due to their structure. Additionally, datasets greatly affect the accuracy of the DL model, and the superiority of one network over another can vary depending on the image quality and specific characteristics of the application datasets.

Approximately two-thirds (69%) of recent studies used publicly available CMR databases for model training [4]. Common public databases include the Heart Database [38], York [39], Sunnybrook [40], LV-2011 [41], and Automated Cardiac Diagnosis Challenge [42]. Regardless of the methods used to segment the LV, most studies have shown a level comparable to that of human experts. Dice scores were often used to assess the agreement between the automated and manual segmentations. A Dice score of 0% indicates no agreement, and a Dice score of 100% indicates perfect agreement. All average values for Dice scores have been >0.85 [4, 10, 11, 12, 43]. For example, Dakua [44], in addition to the weighting function LoDroG, proposed a modified active contour model that integrates local and global image information for LV segmentation in low-contrast magnetic resonance (MR) images. The model showed average Dice scores of 96.1% ± 1.1% for the MICCAI database. However, some of the public databases included single-center data; therefore, the resulting model lacked generalization ability. Moreover, the labeling accuracy in public databases is non-uniform, and the overall evaluation of the segmentation performance may hide the limitations of the poor results for the base and apical regions.

Increasing the number of training data can partially improve the effect of AI. Combining an FCN with a large dataset consisting of 4875 subjects with 93,500 pixelwise annotated images, one method [5] showed high performance and achieved an average Dice metric of 0.88 for the LV myocardium and 0.94 for the LV cavity. Tao *et al*. [13] used CNN with U-Net architecture to automatically identify and segment the LV from multivendor and multicenter CMR images. They found that increasing the variability from the training set improved the test performance outside the datasets. The LV function parameters derived from CNN3 showed a strong correlation (r^2^ ≥ 0.98) and agreement with those obtained by experts. Notably, although a DL-based method trained on a dataset with high variability improves the model’s generalizability, the clinical applicability of the network is unclear owing to the lack of clinical large-scale external validation. Hence, Mariscal-Harana *et al*. [15] used highly structured and controlled databases to train the model and used large external clinical datasets (n = 6888) to verify the effectiveness of the model. Their method resulted in median Dice scores of >91% for LVBP and >83% for LVM, leading to median absolute errors in cardiac biomarkers of <8.4 mL for the LV and <5.9% for EF across all datasets. Extensive verification showed that the tool achieved the human-level accuracy of CMR segmentation for various diseases, vendors and clinical imaging protocols.

Most of the above studies used data from expert manual analysis as the gold standard when analyzing model performance, but there is concern within the field due to inter-observer variability. In a study involving (multiple pathologies with n = 109 patients) CMR scans, the authors evaluated the scan-rescan reproducibility of machine learning algorithm and three clinicians (human). The scan-rescan coefficient of variation was significantly larger for humans compared with that of machines across all parameters except ESV, which was equivalent to that of humans (e.g., LVEF 6.0% vs 4.2%) [14]. The machine learning algorithm had better consistency.

### 2.2 Current Problems

As LV segmentation develops, the technology has matured; however, the following problems remain. (1) The basal and apical myocardium are not accurately identified. (2) At the end of systole, LV segmentation cannot accurately distinguish between the trabeculae and myocardium. (3) The generalizability of the model is insufficient, and the segmentation efficiency differs among diseases, image quality and vendors. In recent years, researchers have made many effective attempts to solve these problems.

Regarding the problem of poor segmentation of the basal myocardium, Penso *et al*. [9] contrasted the segmentation performance of U-Net trained on two criteria: the basal slices where the myocardium surrounded the left ventricle for ≥50% of the slice and all other slices and all slices where the myocardium completely surrounded the left ventricle. They found no significant differences when considering all slices together, but significant differences were present when only basal slices were examined. Xie [45] used the convex hull algorithm to reconnect the two ends of the ring structure of the myocardial segmentation results and remove the false-positive segmentation results near the myocardium to help solve the problem of incomplete basal slice segmentation.

Regarding the problem of low segmentation accuracy in the end-systolic and blurred LV edge information, some DL methods have shown good performance, such as the fine-grained calibrated double-attention convolutional network [16] and the multi-scale multi-skip connection network [17].

Although increasing the training datasets helps improve the model’s generalizability, it has little effect on the model when the number of datasets increases past a certain amount. Additionally, as the datasets increase, the manual labeling workload increases; thus, the potential variability increases. Generative adversarial networks (GANs) offer a promising avenue for addressing data scarcity in medical imaging and enhancing the generalizability of segmentation models through image synthesis. A recent study [46] explored the usability of synthesized short-axis CMR images generated using a segmentation-informed conditional GAN. Additional integration of real and synthetic datasets during the training process notably enhanced the segmentation performance, with improvements reaching 4% for the Dice score and 40% for Hausdorff distance across diverse datasets sourced from different sites and scanners. Deep mining of information from smaller datasets is another new research direction. Guo *et al*. [47] developed a globally optimal label fusion (GOLF) algorithm and an uncertainty-guided coupled continuous kernel cut (ugCCKC) algorithm and further integrated GOLF and ugCCKC into a DL ensemble segmentation framework. Using relatively modest datasets (consisting of 5–10 subjects) with sparse annotations (ranging from 5%–25% of labeled slices), the DL algorithm achieved impressive Dice scores of 0.871–0.893 for LVM and 0.933–0.959 for LVBP on the LVQuan dataset. These findings indicate the potential of the developed methodologies to streamline DL integration into both research and clinical CMR imaging workflows.

Recently, the heat waves of diffusion models have poured into medical imaging. Diffusion models have been found to be useful in a wide variety of areas, including image generation, image super-resolution, image segmentation, classification, and anomaly detection [48]. Kim and Ye [49] proposed a diffusion deformable model for four dimensional (4D) image generation. The method generated realistic deformed volumes along the trajectory between the cardiac diastolic and systolic phases, which outperforms the existing registration-based models. Rahman *et al*. [50] creatively proposed to use a diffusion model to learn collective expert diagnostic opinions for more effective medical image segmentation. It is possible to fully exploit the advantages of the diffusion model in CMR analysis to solve the current problems of LV segmentation, further improve the robustness of the model, and promote the transformation of the model into a truly valuable tool in clinical practice.

## 3. Regional Function Assessment

The quantitative parameters of cardiac function obtained by accurate LVM and LVBP segmentation enable global evaluation of the heart. However, comprehensive analysis of the heart is crucial for a detailed evaluation of local cardiac function. Specifically, the LVM is divided into 17 segments according to the guidelines [3], and the myocardial thickness and myocardial motion of each segment are further evaluated [51, 52, 53, 54, 55]. To fully automate the entire CMR analysis, a series of AI algorithms have been generated. The specific information is as follows.

### 3.1 Seventeen Cardiac Segments

The American Heart Association recommends dividing the left ventricle into three equivalent parts along the long axis of the heart: the base, mid-cavity, and apex, then further subdividing them into 17 segments using the anterior and inferior right ventricular (RV) insertion points [56]. A fully automatic hybrid framework was proposed that detected mitral valve points on long-axis CMR and RV insertion points on short-axis CMR [18]. The framework achieved a final average error of 2.87 mm for the mitral valve points and an average error of 3.64 mm for the RV insertion points. Xue *et al*. [19] also developed a network to detect these landmarks; however, their study included cine sequences as well as LGE and T1 mapping. Detection rates were high on the short-axis images, with successful rates of 96.6% for cine, 97.6% for LGE, and 98.7% for T1 mapping. The Euclidean distances between the landmarks assigned by the model and those assigned manually ranged from 2–3.5 mm for various points, demonstrating close agreement between the landmarks derived from the model and the manually assigned labels. Identifying key landmarks by dividing the myocardium into 17 segments is a prerequisite for disease localization. The current related AI research is minimal, but it may become an important new direction.

### 3.2 Myocardial Thickness

Changes in myocardial thickness differ among disease states. Patients with hypertensive heart disease or hypertrophic cardiomyopathy (HCM) usually show thickening of the LV wall, whereas patients with dilated cardiomyopathy or old myocardial infarction usually show thinning of the LV wall. Judging the thickening or thinning of the ventricular wall helps to differentially diagnose these diseases. Manually measuring myocardial thickness is tedious and repetitive, and AI can simplify the tedious work. Clinicians must calculate only the vertical distance between the endocardium and epicardium of the left ventricle by the algorithm to obtain the myocardial thickness value. LV segmentation is a more mature development, and automation for measuring myocardial thickness lacks technical difficulties. One goal of the MICCAI challenge was to quantify regional wall thicknesses. The datasets provided in this challenge enabled generating a series of intelligent methods for automatically measuring myocardial thickness [57, 58, 59]. Unfortunately, this challenge was mainly for the middle myocardium, and whether it can be used for the whole left ventricle requires further verification. Khalid *et al*. [60] developed a novel framework of three-dimensional (3D) personalized LV modeling and a wall thickening assessment algorithm based on sphere fitting to evaluate regional cardiac wall thickening dysfunction from base to apex across all cardiac phases. Moreover, Augusto *et al*. [20] proposed an automated ML tool for maximum wall thickness measurement in HCM. They found that ML precision was superior and a significantly lower coefficient of variation than for all experts (4.3% vs 5.7–12.1% across experts).

### 3.3 Myocardial Motion

To comprehensively assess LV function, the myocardial motion must be assessed to diagnose cardiovascular diseases. DL can simultaneously achieve myocardial segmentation and motion assessment [21], and some studies have demonstrated that the performance of DL algorithms in identifying myocardial wall motion abnormalities at rest in patients with ischemic heart disease is comparable to that of subspecialty radiologists [22]. Presently, the common methods of evaluating LV motion include mainly feature tracking and deformable image registration (DIR).

Feature tracking methods typically begin by segmenting the ventricular wall and subsequently tracking the displacement of feature points, such as the myocardial contours. Throughout this process, the primary challenge is accurately estimating the correspondence between sampled points on the contours at various time points. Remme *et al*. [61] manually tracked a sparse number of fiducial markers through the cardiac cycle and combined the results with a parameter distribution model of LV deformations to estimate LV motion. Wu *et al*. [24] proposed a feature point descriptor using FCN to extract features of points from short-axis cine MR images. Introducing the FCN feature descriptor to a graph-matching algorithm improved the correspondence accuracy between points located on the LVM boundary to estimate cardiac motion.

DIR algorithms can establish an anatomical correspondence between a pair of images and have been used to assess myocardial motion [23, 25, 26, 62, 63, 64]. Study has shown that DIR algorithms allow for assessing myocardial deformation with reduced variability and superior reproducibility compared with that of MR feature tracking [62]. For example, Morales *et al*. [23] proposed an unsupervised learning-based approach for deformable 3D cardiac MR image registration. The method achieved a median dice similarity coefficient (DSC) of 0.77 for pediatric data, which was higher than or similar to all other methods mentioned herein. Importantly, most of the proposed methods focus on recovering deformations between individual pairs of images, thus failing to capture temporal dependencies. To address these limitations, Zakeri *et al*. [25] proposed a novel probabilistic spatiotemporal registration framework, with the advantages of offering analytical pixelwise motion uncertainty estimation across a cardiac cycle and of being a motion generator.

Owing to a lack of efficiency and accuracy, many methods developed to automatically estimate myocardial motion have not been widely adopted in clinical settings. A new approach was recently proposed to address this problem. Ye *et al*. [65] proposed a novel bidirectional unsupervised diffeomorphic registration network to estimate the interframe motion and residual Lagrangian motion. Although the method was heavily validated on in-house and public datasets composed of different imaging modalities, larger clinical datasets are needed to verify their effectiveness.

### 3.4 Myocardial Fibrosis

Many studies have shown that myocardial fibrosis (MF) quantified by LGE affects the long-term prognoses of patients with heart disease [66, 67, 68, 69, 70]. Accurately identifying and quantifying MF requires a high professional standard for clinicians. Variation among clinicians and analysis of core laboratories reduces the reproducibility of MF quantification and hinders its clinical utility [71, 72]. Automatic LGE image analysis has been proposed to mitigate the time for manual assessment and improve consistency [27, 28, 29, 30, 73, 74, 75]. Most DL algorithms used CNN-based networks, and the performance of 3D CNN was better than that of two dimensional (2D) CNN. The 3D CNN-based quantification strongly correlated with manual quantification of scar volume (r = 0.82–0.99) and %LGE (r = 0.90–0.97) for all sites and vendors [28]. However, automatic segmentation is imprecise when adjacent non-myocardial tissue is near the LGE signal intensity, such as that of hyper-enhanced BP or adipose tissues [76]. When this happens, cine sequences are usually needed to accurately identify the myocardial borders. Fahmy *et al*. [31] developed a DL model for combining LGE and cine images, which showed better agreement with manual quantification of LGE scar burden than did CNN without LGE-cine fusion.

In addition to judging with or without LGE, classification of LGE is also crucial for disease diagnosis. Ischemic cardiomyopathy often presents with subendocardial LGE, while delayed enhancement of nonischemic cardiomyopathy is restricted to the middle or subendocardium [3]. Deep neural network architecture for automatic detection of LGE patterns have been developed [77]. The three networks (GoogLeNet, AlexNet, and ResNet-152 CNNs) proposed showed good accuracy: 79.5% (1592/1995 images), 78.9% (1574/1995 images), and 82.1% (1637/1995 images), respectively.

Notably, LGE imaging requires injection of gadolinium contrast agents (GBCAs), which is not applicable to some patients, such as those with contrast allergies and nephrogenic fibrosis [78]. Several techniques have been developed to analyze MF without using GBCAs, including feature extraction from cine sequences, native T1 mapping [32], and magnetization transfer contrast [79]. This review focuses on evaluating MF based on cine sequences. Several studies have been reported on fully automated segmentation of MF from cine CMR images using DL-based methods [33, 80, 81]. These algorithms are validated by comparing them to the ground truths manually segmented from corresponding LGE CMR images. Additionally, some researchers have developed cine imaging-based models to identify patients at high risk of fibrosis, thus avoiding unnecessary GBCA injection in patients without fibrosis, which saves medical resources and benefits patients. A multicenter study of 1099 patients with HCM established an XGBoost ML model that combined regional wall thickness, thickening and radiomic features [34]. The model yielded an area under the curve of 0.83. Of 82 patients identified as lacking fibrosis by the model in the validation set, 73 (89%) were correctly classified as true negatives, thus potentially avoiding the need for enhanced CMR scans. A similar study combined the CMR model obtained from the multivariable regression analysis with the radiomic features and showed better results, with an area under the curve of 0.898 [82]. Most studies focus on a specific cardiovascular disease, and its application to others still requires extensive validation, and quantitative evaluation of myocardial fibrosis is lacking.

## 4. Quality Control

In clinical imaging, variability in image quality, presence of artifacts, and unexpected anatomical variations (not encountered during training) are inevitable, potentially leading to substantial errors if subjected to automatic analysis. Therefore, robust quality control protocols are essential for identifying potential erroneous outputs, serving as a prerequisite for successful implementation of AI algorithms in clinical settings [83]. It helps to improve the image quality, highlight the structure and detail by enhancing the contrast of the data, enabling models to extract crucial features from cardiac images more accurately. This further improves the accuracy and robustness.

Quality control can be divided into the pre-analysis control of image quality and a post-analysis control of the quality of the output parameters [15, 35, 36, 37, 84, 85, 86, 87, 88, 89]. The pre-analysis control focused on detection of motion artifacts owing to inconsistent breath-holding, mistriggering or arrhythmias, noise reduction and automatic view planning [35, 37, 84, 85]. Oksuz *et al*. [35] proposed a novel k-space-based training data augmentation approach that could detect 2D+time short-axis images with motion artifacts in <1 ms. Zhang *et al*. [84] proposed a novel semi-supervised method to check the coverage of LV by using generative adversarial networks. The post-analysis control focused on detection of the presence of missing slices, segmentation quality assessment and uncertainty estimation [36, 37, 86, 87, 88]. Joyce *et al*. [86] proposed a differentiable volumetric mesh voxelization technique based on deformation of a shape model, which could correct for slice misalignment and was robust enough for incomplete and inaccurate input data. Albà *et al*. [88] used statistical, pattern and fractal descriptors in a random forest classifier to detect incorrect cardiac segmentations to be corrected or removed from subsequent analysis. Some researchers have used clinical knowledge of the basic equivalence of left and right ventricular SV to detect cardiac function with incorrect output [37].

## 5. Conclusions and Future Outlooks

**Fig. 2.**
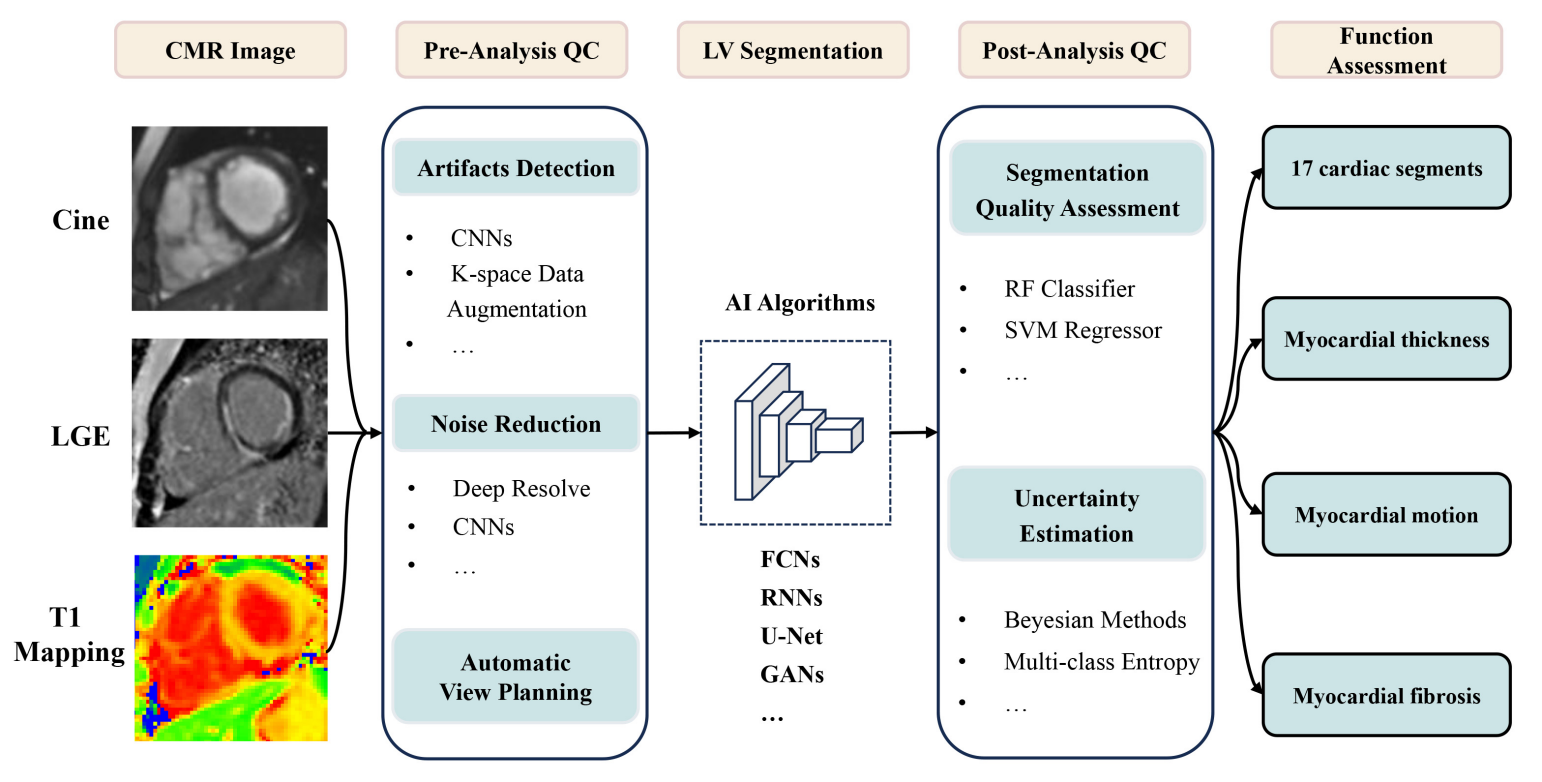
**Application of AI in left ventricle analysis on CMR**. CMR, cardiovascular magnetic resonance; LGE, late gadolinium enhancement; QC, quality control; CNNs, convolutional neural networks; LV, left ventricular; AI, artificial intelligence; FCNs, fully convolutional networks; RNNs, recurrent neural networks; GANs, generative adversarial networks; RF, random forest; SVM, support vector machine.

The feasibility of applying AI to LV functional analysis on CMR has been widely demonstrated, as shown in Fig. 2; however, the evaluation of numerous algorithms remains heterogeneous, and future clinical trials are needed to determine the effectiveness of algorithms in clinical practice. Additionally, the left heart segmentation algorithm is mature, and other aspects, such as intelligent analysis of LGE, still face great challenges. In the future, how to reduce the workload of human annotation and increase the accuracy of training data deserves our attention. It has great advantage in end-to-end neural network, which can directly output target outcomes. It is expected to integrate multimodal information at the clinical level and perform in-depth phenotyping using multimodal large models. With the rapid development of discriminative and generative models, it will play an important role in the diagnosis and risk assessment of cardiovascular diseases.

## Abbreviations

CMR, cardiac magnetic resonance; AI, artificial intelligence; EDV, end-diastolic volume; ESV, end-systolic volume; SV, stroke volume; CO, cardiac output; EF, ejection fraction; LGE, late gadolinium enhancement; LVM, left ventricular myocardium; BP, blood pool; DL, deep learning; ML, machine learning; CNN, convolutional neural network; FCN, fully convolutional network; MF, myocardial fibrosis.
